# Australia’s east coast humpback whales: Satellite tag-derived movements on breeding grounds, feeding grounds and along the northern and southern migration

**DOI:** 10.3897/BDJ.11.e114729

**Published:** 2023-12-11

**Authors:** Virginia Andrews-Goff, Nick Gales, Simon J Childerhouse, Sarah M Laverick, Andrea M Polanowski, Michael C Double

**Affiliations:** 1 Australian Antarctic Division, Department of Climate Change, Energy, the Environment and Water, Hobart, Australia Australian Antarctic Division, Department of Climate Change, Energy, the Environment and Water Hobart Australia; 2 Department of Climate Change, Energy, the Environment and Water, Hobart, Australia Department of Climate Change, Energy, the Environment and Water Hobart Australia; 3 Environmental Law Initiative, Wellington, New Zealand Environmental Law Initiative Wellington New Zealand; 4 Blue Planet Marine, Canberra, Australia Blue Planet Marine Canberra Australia

**Keywords:** satellite telemetry, breeding stock E1, conservation, management, foraging, Antarctica, baleen whale, Southern Ocean, *Megapteranovaeangliae*

## Abstract

**Background:**

Satellite tags were deployed on 50 east Australian humpback whales (breeding stock E1) between 2008 and 2010 on their southward migration, northward migration and feeding grounds in order to identify and describe migratory pathways, feeding grounds and possible calving areas. At the time, these movements were not well understood and calving grounds were not clearly identified. To the best of our knowledge, this dataset details all long-term, implantable tag deployments that have occurred to date on breeding stock E1. As such, these data provide researchers, regulators and industry with clear and valuable insights into the spatial and temporal nature of humpback whale movements along the eastern coastline of Australia and into the Southern Ocean. As this population of humpback whales navigates an increasingly complex habitat undergoing various development pressures and anthropogenic disturbances, in addition to climate-mediated changes in their marine environment, this dataset may also provide a valuable baseline.

**New information:**

At the time these tracks were generated, these were the first satellite tag deployments intended to deliver long-term, detailed movement information on east Australian (breeding stock E1) humpback whales. The tracking data revealed previously unknown migratory pathways into the Southern Ocean, with 11 individuals tracked to their Antarctic feeding grounds. Once assumed to head directly south on their southern migration, five individuals initially travelled west towards New Zealand. Six tracks detailed the coastal movement of humpback whales migrating south. One tag transmitted a partial southern migration, then ceased transmissions only to begin transmitting eight months later as the animal was migrating north. Northern migration to breeding grounds was detailed for 13 individuals, with four tracks including turning points and partial southern migrations. Another 14 humpback whales were tagged in Antarctica, providing detailed Antarctic feeding ground movements.

Broadly speaking, the tracking data revealed a pattern of movement where whales were at their northern limit in July and their southern limit in March. Migration north was most rapid across the months of May and June, whilst migration south was most rapid between November and December. Tagged humpback whales were located on their Antarctic feeding grounds predominantly between January and May and approached their breeding grounds between July and August. Tracking distances ranged from 68 km to 8580 km and 1 to 286 days. To the best of our knowledge, this dataset compiles all of the long-term tag deployments that have occurred to date on breeding stock E1.

## Introduction

Humpback whales are globally distributed, occupying each of the ocean basins (Jackson et al. 2014). Like most rorqual whale species, humpback whales were targeted by the industrial whaling industry, with around 220,000 humpback whales killed in the Southern Hemisphere between 1904 and 1973 (Jackson et al. 2015). In order to manage humpback whale stocks, the International Whaling Commission assigned seven Southern Hemisphere breeding stocks (A-G) and six Southern Ocean feeding areas (Donovan 1991). The two breeding stocks (D and E1) that move along Australia’s west and east coasts annually were likely reduced to just hundreds of individuals each when industrial and illegal whaling ceased (Chittleborough 1965, Bannister and Hedley 2001). However, despite the devastation caused by whaling, Australia’s humpback whales have demonstrated a remarkable population recovery and, in 2022, the Australian Government removed their threatened species listing under the Environment Protection and Biodiversity Conservation Act 1999. Australia’s eastern population of humpback whales was estimated to number 24,545 in 2015, with full recovery of the population expected to occur in 2016 (Noad et al. 2019). A contemporary population estimate for Australia’s western population of humpback whales is lacking. However, in 2008, the population was conservatively estimated to be 17,810 individuals (Hedley et al. 2011).

Humpback whales undertake the longest mammalian migration on the planet (Rasmussen et al. 2007), moving seasonally between their winter breeding/calving grounds located in tropical and subtropical waters to their high-latitude summer feeding grounds, with the exception of the resident Arabian Sea population (Mikhalev 1997). Whilst some of this migration occurs along populated coastline, which facilitates the spatial and temporal monitoring of movements (for example, Noad et al. 2019 and Pirotta et al. (2020)), the majority of movement is far removed from land (for example, Andrews-Goff et al. (2018), Bestley et al. (2019)). Satellite tags are the primary technology used to detail movement over biologically relevant time scales (Dingle 2014) and are especially essential to determine long-term, large-scale, detailed movements. Satellite tag-derived data are critical for identifying habitat use (Reisinger et al. 2021), overlap with threats (Weinstein et al. 2017) and novel behaviour (Garrigue et al. 2015) and is an essential tool for conservation and management of an animal that spends very little time at the surface (Nowacek et al. 2016) and in remote areas with no survey effort (Mate et al. 2007).

We present here a dataset detailing the satellite tag-derived movements of 50 humpback whales from Australia’s eastern breeding stock E1. To the best of our knowledge, this dataset compiles all of the long-term tag deployments (type C implantable satellite tags; Andrews et al. (2019)) that have occurred to date on breeding stock E1. The dataset details movements on coastal breeding grounds, along northern and southern migrations and on Antarctic feeding grounds. These tracks have been compiled in their raw form, with a basic speed distance angle filter applied and also as a state space model output that accounts for Argos location error. These data provide researchers, regulators and industry with clear and valuable insights into the spatial and temporal nature of humpback whale movements along the eastern coastline of Australia. Managing and protecting species that cross ocean basins and jurisdictions is a challenge (Asaro 2012, Geijer and Jones 2015, Miller et al. 2018). As humpback whales navigate an increasingly complex habitat undergoing various development pressures and anthropogenic disturbances (Bolin et al. 2020, Indeck et al. 2021, Mayaud et al. 2022), as well as a marine environment changing under a shifting climate regime (Tulloch et al. 2019, Pallin et al. 2023), this dataset may also provide valuable baseline data.

## General description

### Purpose

Satellite tags were deployed on humpback whales on their southward migration, northward migration and feeding grounds in 2008, 2009 and 2010 to describe migratory pathways and movements on Antarctic feeding grounds and to identify possible calving areas. At the time, these movements were not well understood and calving grounds were not clearly identified.

### Additional information

This dataset revealed the following key results:

- Supplemental feeding by breeding stock E1 humpback whales in temperate waters on their southern migration (Gales et al. 2009) despite the fact that humpback whales were generally assumed to only feed on their Antarctic feeding grounds;
- A previously unknown migratory pathway departing the Australian coastline in an eastward direction towards the western coastline of New Zealand's South Island Te Waipounamu and then on to Antarctica (Gales et al. 2009);
- Migration in a westerly direction across the Bass Strait to forage in IWC Management Area IV by one individual. Whilst breeding stock E1 humpback whales generally forage in IWC Management Area V, this whale travelled to IWC Management Area IV, mixing with the humpback whales that migrate south along the Western Australian coastline (breeding stock D; Gales et al. (2009));
- The northern extent of the migratory pathway for breeding stock E1 humpback whales is located within the southern Great Barrier Reef (Gales et al. 2010). These tracking data supported designation of an important wintering area off Proserpine and Mackay (19.5°S to 21.5°S; Smith et al. (2012));
- Proved that it was possible to attach satellite tags to humpback whales located in high latitude seas (Gales 2010). The Antarctic foraging habitat of these whales tagged in IWC Management Area V (where breeding stock E1 humpback whales aggregate, Constantine et al. (2014)) is associated with the marginal ice zone. Key predictors of inferred foraging behaviour include distance from the ice edge, ice melt rate and variability in ice concentration two months prior to arrival (Andrews-Goff et al. 2018).

## Project description

### Title

Satellite tag-derived movements of Australia’s eastern humpback whale population, breeding stock E1

### Personnel

Nick Gales, Sarah Laverick, Mike Double, Simon Childerhouse, Dave Paton, Curt Jenner

### Study area description

Satellite tags were deployed on whales in the following locations:

- Eden, southern NSW (Australia), October 2008: whales were tagged off Eden during their southern migration.
- Evans Head, northern NSW (Australia), June and July 2009: whales were tagged off Evans Head during their northern migration.
- East Antarctica, February 2010: whales were tagged on their feeding grounds within IWC Management Area V.
- Sunshine Coast, QLD (Australia), October 2010: whales were tagged off the Sunshine Coast during their southern migration.

The satellite-tagged humpback whales ranged widely from the tropical waters of the Great Barrier Reef (16°S) to the polar waters of Antarctica (70°S). The tracked whales moved through a region spanning a longitudinal range of 83° (between 101°E and 176°W). When on their Antarctic feeding grounds, whales moved through IWC Management Areas IV (70°E to 130°E; Donovan (1991)) and V (130°E to 170°W; Donovan (1991)) with the majority of movements concentrated in Area V. When migrating along the Australian coastline, movements were predominantly restricted to over the continental shelf and over sandy substrate.

The datasets described here are available in the Movebank Data Repository, https://doi.org/10.5441/001/1.294 (Andrews-Goff et al. 2023).

### Funding

These satellite tag deployments were undertaken by the Australian Marine Mammal Centre funded by the Commonwealth Environment Research Fund (CERF) and then the Australian Government’s International Whale and Marine Mammal Conservation Initiative (IWMMCI), as well as the Australian Antarctic Division.

## Sampling methods

### Study extent

Satellite tags were deployed on humpback whales located off east Australia (2008, 2009, 2010) and in east Antarctica (2010). Locations were transmitted via the Argos satellite system and processed to account for erroneous locations and the spatial error associated with Argos locations.

### Sampling description

**Satellite tag deployment**

Type C implantable satellite tags (Andrews et al. 2019) were deployed on humpback whales in good body condition using a modified version of the Air Rocket Transmitter System (ARTS), Restech (Heide-Jorgensen et al. 2001) and a purpose-designed projectile carrier at a pressure of 7–12 bar. Deployment details are given within the Data Resources package, with additional information capturing tracking duration, deployment location, behaviour and type of movement described in Table 1. The satellite tag employed was comprised of a stainless-steel cylindrical housing containing a location-only SPOT-5 transmitter manufactured by Wildlife Computers (Redmond, Washington, USA) or a Kiwisat 202 Cricket (Sirtrack, Havelock North, New Zealand) plus an anchor section (320 mm in length). The tag was designed to penetrate the skin and blubber with retention via a spring-loaded, articulated anchor and passively deployed petals. This articulated design is now superseded. Deployment of the tag using the ARTS was aided by a purpose-designed projectile carrier, often referred to as a ‘rocket’ or ‘sabot’. Retention teeth on the projectile carrier are gripped to a metal ring secured to the end of the tag. When the tag came into contact with the whale, the rapid deceleration of the tag and the projectile carrier withdrew the retention teeth, releasing the projectile carrier. The metal ring then fell off in time to reduce the drag of the tag. Satellite tags were sterilised with ethylene oxide prior to deployment and implanted up to a maximum of 290 mm into the skin, blubber, interfacial layers and outer muscle mass of the whale. Each tag was deployed from the bow-sprit of a purpose-built 6.3 m aluminium Naiad RHIB and was positioned high and forward on the body. Satellite tags transmitted data via the Argos satellite system once the tag was immersed in salt water, activating the salt water switch. Tags were programmed to transmit at various duty cycles to extend battery life and tag deployment duration. Tag transmissions were relayed to processing centres to calculate the transmitter’s location by measuring the Doppler Effect on transmission frequency. Transmitted data were processed using least squares analysis and each location was assigned an estimated error and one of seven associated location classes (LC; see CLS (2023)). Briefly, LC 3 has an estimated error of 250 m, LC 2 has an estimated error between 250 and 500 m and LC 1 has an estimated error between 500 and 1500 m. LC 0 has an open-ended error of 1500 m, whilst LC A and B have no accuracy estimation and LC Z is an invalid location. Tags ceased transmitting when they were either naturally shed, damaged, experienced sensor fouling or the battery was exhausted.

Upon tag deployment, a small amount of skin and blubber was simultaneously collected for genetic analyses. These were collected using a biopsy dart fired from a modified 0.22 Paxarms system (Krutzen et al. 2002). Biopsy samples were stored in 70% ethanol and DNA subsequently extracted using a Tissue DNA purification kit for the Maxwell 16 DNA extraction robot (Promega Corporation). The sexes of the tagged whales were determined using a 5′ exonuclease assay of the polymorphisms in the sex-linked Zinc Finger genes as described by Morin et al. (2005). This research was conducted using non-lethal methods that are designed to learn about whales without harming them. The research was approved by the Australian Antarctic Ethics Committee (under Australian Antarctic Science Project 2941) and complied with all relevant permits, including the Australian Government Environment Protection and Biodiversity Conservation Act Cetacean Permit (2007-0007).

### Quality control

**Argos data processing to remove erroneous locations and account for Argos location error**

Using the raw Argos tracking dataset and for all tracks containing > 5 Argos locations, we accounted for the spatial error associated with Argos locations by fitting a correlated random walk state-space model to generate a location estimate at each observed location time (fit_ssm function in the aniMotum package; Jonsen et al. (2023)) within R (R Core Team 2023). Within this state-space model, we applied the sdafilter function, which is an algorithm based on swimming speed, distance between successive locations and turning angles (sdafilter function in the Argosfilter package; Freitas et al. (2008)) to remove unlikely position estimates (speed of 10 ms^−1^, spike angles of 15° and 25°, spike lengths of 2,500 m and 5,000 m). Individual tracks were split into track segments for processing where data gaps exceeded 24 hours.

## Geographic coverage

### Description

**Figure 1.**
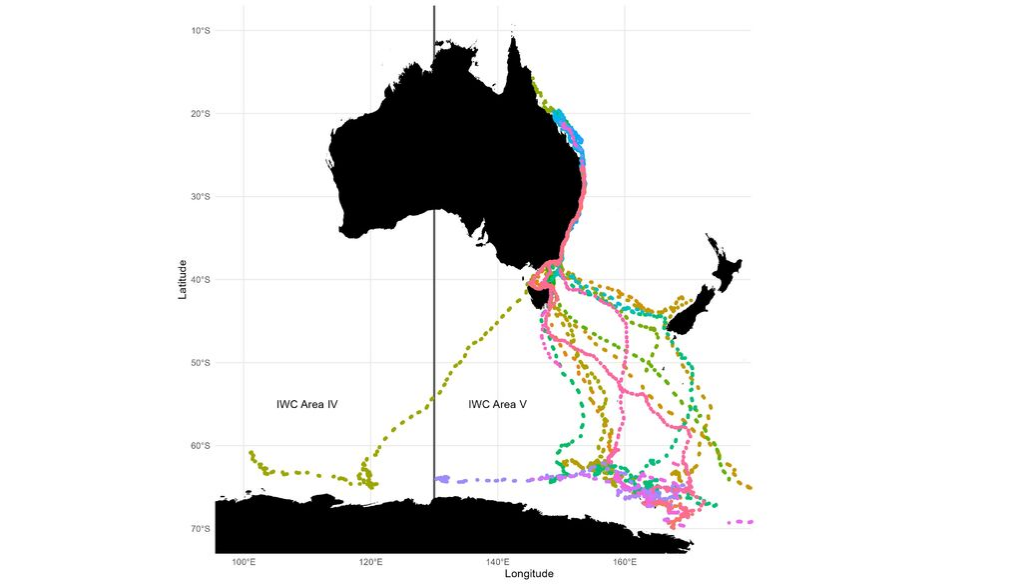
State-space model location estimates for 48 east Australian (breeding stock E1) humpback whales. Two tracks contained < 5 Argos locations so were not included in the state-space model. The boundary between IWC Management Areas IV and V is depicted by the vertical black line at 130°E.

The geographic range of the bulk of the dataset is along the east coast of Australia and broadly through the east Antarctic sector of the Southern Ocean, concentrating in IWC Management Area V (Fig. 1). The tracking data captured various geographic ranges of movement, including southern migration along the east coast of Australia into the Southern Ocean (n = 10), southern migration towards New Zealand (n = 4) and southern migration via New Zealand into the Southern Ocean (n = 1). Six of the tags only transmitted coastal movement on the southern migration. Of these, one tag transmitted a partial southern migration to approximately 50°S, then ceased transmissions only to begin transmitting eight months later at approximately 37°S as the animal was migrating north. Northern migration to breeding grounds was also captured (n = 13), including turning points and partial southern migrations (n = 4). Movement restricted solely to Antarctic feeding grounds was captured by another 14 tracks. Tracking distances ranged from 68 km to 8,580 km (Table 1).

### Coordinates

-70.0 and -15.7 Latitude; -175.2 and 101.1 Longitude.

## Taxonomic coverage

### Description

This dataset focuses exclusively on the humpback whale – *Megapteranovaeangliae* (Borowski, 1781) (Balaenopteridae, order Artiodactyla), which is categorised as Least Concern in the IUCN Red List (Cooke 2018). This dataset details the east Australian humpback whale breeding stock/population E1. The Australian Government categorises this population as vulnerable.

### Taxa included

**Table:** 

Rank	Scientific Name	Common Name
species	*Megapteranovaeangliae*	Humpback whale

## Temporal coverage

**Data range:** 2008-10-24 – 2011-7-27.

### Notes

**Figure 2.**
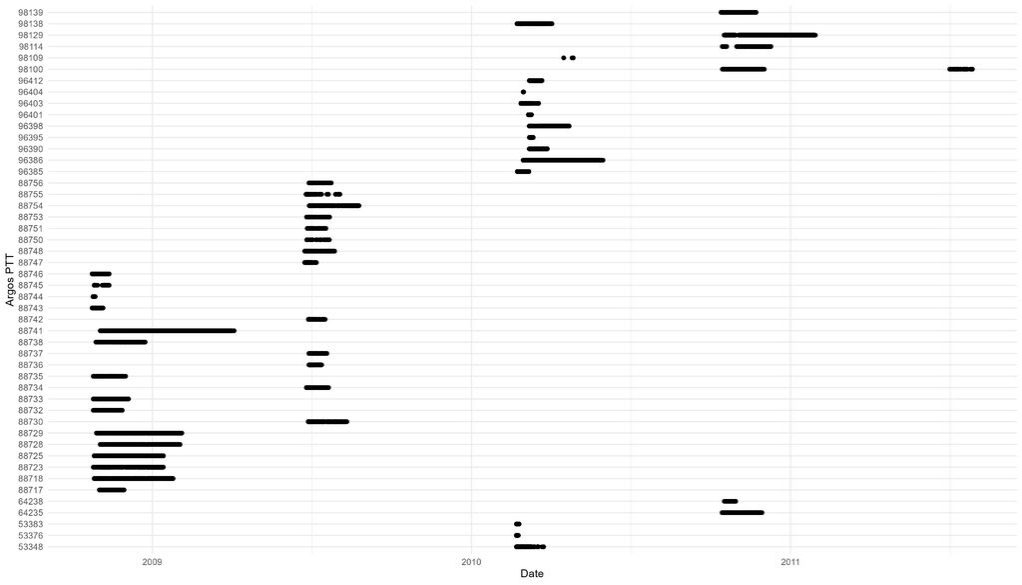
Deployment duration (x axis) for each of the deployed satellite tags (unique Argos PTT on the y axis). Deployments span 2009, 2010 and 2011. Satellite tags transmitted locations continuously (for example, 88741) or sometimes intermittently (for example, 88755).

Tags transmitted data over 1 to 286 days; however, not all tags transmitted continuously (Fig. 2).

**Figure 3.**
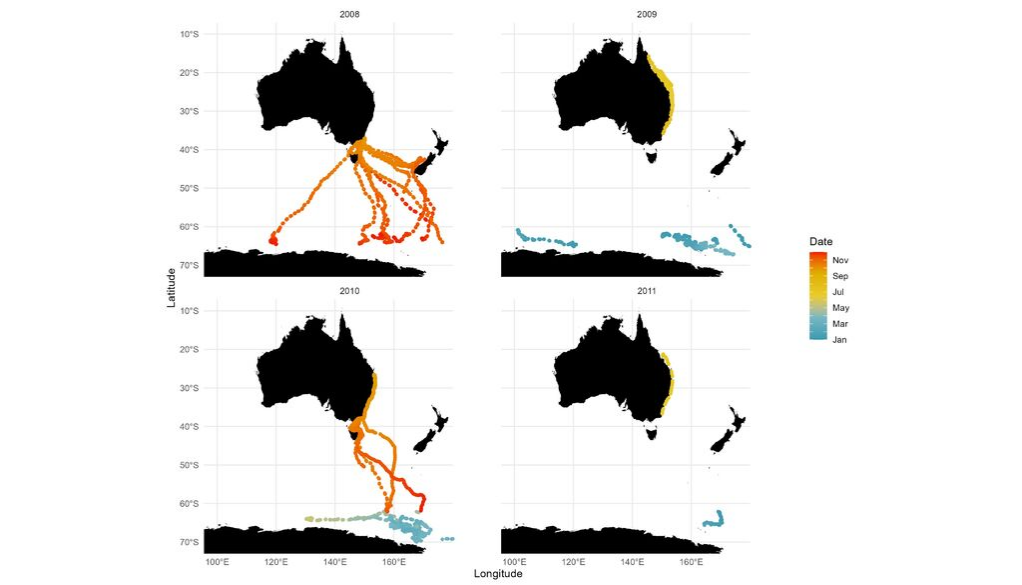
State-space model location estimates generated by satellite tagged east Australian (breeding stock E1) humpback whales in each year and coloured according to month.

Tags transmitted locations for each month of the year with the exception of September (Table 2; Fig. 3). The temporal pattern of movement can be broadly described by assessing mean latitude against month, acknowledging that there is individual variability in the dominant direction of travel in each month. On average, tagged humpback whales were at their northern limit in July and their southern limit in March. Migration north was most rapid across the months of May and June, with mean latitude in May at 64.0°S and mean latitude in June at 27.1°S. Migration south was most rapid between November (mean latitude of 44.6°S) and December (mean latitude of 58.7°S). Tagged humpbacks were located on their Antarctic feeding grounds predominantly between January and May and approach their breeding grounds between July and August (noting that there are no location data for September).

## Usage licence

### Usage licence

Other

### IP rights notes

CC BY: This licence allows reusers to distribute, remix, adapt and build upon the material in any medium or format, so long as attribution is given to the creator. The licence allows for commercial use.

## Data resources

### Data package title

East Australian (breeding stock E1) humpback whale tracking data – satellite tag-derived Argos locations and associated information, reference data detailing tag deployments and state-space model location estimates that provide a dataset that accounts for erroneous locations and Argos location error. Datasets are freely available and are published in the Movebank data repository and the Australian Antarctic Data Centre.

### Resource link

https://www.movebank.org/cms/webapp?gwt_fragment=page=studies,path=study3030068329

### Number of data sets

2

### Data set 1.

#### Data set name

Movements of Australia's east coast humpback whales

#### Data format

csv

#### Download URL

https://www.movebank.org/cms/webapp?gwt_fragment=page=studies,path=study3030068329

#### Description

This file contains all Argos locations generated by satellite tags deployed on 50 humpback whales, as detailed in Table 1 and the reference data within Data Resources. Using the raw Argos tracking dataset, but only for tracks containing > 5 locations (n = 48), we accounted for the spatial error associated with Argos locations by fitting a correlated random walk state-space model to generate a location estimate at each observed location time. Within this state-space model, we applied the sdafilter to remove unlikely position estimates (speed of 10 ms^−1^, spike angles of 15° and 25°, spike lengths of 2500 m and 5000 m). The associated state-space model locations for 48 humpback whales are also contained within this file and are identified within the columns 'comments' ('state-space model location estimate – see citation for details') and 'modelled' ('TRUE').

**Data set 1.** 

Column label	Column description
event-id	An identifier for the set of values associated with each event. A unique event ID is assigned to every time-location record.
visible	Determines whether an event is visible on the Movebank map.
timestamp	The date and time corresponding to each location estimate. Format: yyyy-MM-dd HH:mm:ss.SSS; units/time zone: UTC.
location-long	The geographic longitude of the location as estimated by the sensor. Positive values are east of the Greenwich Meridian, negative values are west of it. Units: decimal degrees, WGS84 reference system.
location-lat	The geographic latitude of the location as estimated by the sensor. Units: decimal degrees, WGS84 reference system.
algorithm-marked-outlier	Identifies events marked as outliers using a user-selected filter algorithm in Movebank. Outliers have the value TRUE. Information about how outliers were defined provided in 'outlier comments' in the associated reference data.
argos:lat1	Argos' primary geographic latitude location estimate. Units: decimal degrees, WGS84 reference system.
argos:lat2	Argos' alternate geographic latitude location estimate. Units: decimal degrees, WGS84 reference system.
argos:lc	The location class retrieved from Argos, Argos diagnostic data. Classes are based on the type of location (Argos Doppler Shift or GPS) and the number of messages received during the satellite pass. Location classes in order of decreasing accuracy are G (GPS), 3, 2, 1, 0, A, B and Z (definition from Argos User's Manual V1.6.6, 2016).
argos:location-algorithm	The processing algorithm used by Argos to estimate locations using Doppler shift.
argos:lon1	Argos' primary geographic longitude location estimate. Positive values are east of the Greenwich Meridian, negative values are west of it. Units: decimal degrees, WGS84 reference system.
argos:lon2	Argos' alternative geographic longitude location estimate. Positive values are east of the Greenwich Meridian, negative values are west of it. Units: decimal degrees, WGS84 reference system.
comments	Additional information - identifies state-space model locations.
modelled	Identifies locations that are modelled (marked as TRUE).
sensor-type	The type of sensor with which data were collected. Argos Doppler shift = The sensor location is estimated by Argos using Doppler shift.
individual-taxon-canonical-name	The scientific name of the species on which the tag was deployed, as defined by the Integrated Taxonomic Information System (ITIS).
tag-local-identifier	An identifier for the tag.
individual-local-identifier	An individual identifier for the animal.
study-name	The name of the study in Movebank.

### Data set 2.

#### Data set name

Movements of Australia's east coast humpback whales-reference-data

#### Data format

csv

#### Download URL

https://www.movebank.org/cms/webapp?gwt_fragment=page=studies,path=study3030068329

#### Description

Reference data detailing satellite tag deployments on Australia's east coast humpback whales (n = 50).

**Data set 2.** 

Column label	Column description
tag-id	A unique identifier for the deployment of a tag on animal.
animal-id	An individual identifier for the animal.
animal-taxon	The scientific name of the species on which the tag was deployed, as defined by the Integrated Taxonomic Information System (ITIS, www.itis.gov).
deploy-on-date	The timestamp when the tag deployment started. Format: yyyy-MM-dd HH:mm:ss.SSS units: UTC.
deploy-off-date	The timestamp when the tag deployment ended. Format: yyyy-MM-dd HH:mm:ss.SSS units: UTC.
animal-group-id	The name or identifier for an associated group, in this case the breeding stock identity.
animal-life-stage	The age class or life stage of the animal at the beginning of the deployment. Can be years or months of age or terms such as 'adult', 'subadult' and 'juvenile/calf'.
animal-sex	The sex of the animal. Allowed values are m = male; f = female; u = unknown.
attachment-type	The way a tag is attached to an animal; 'implant' = the tag is placed under the skin of the animal.
deploy-on-latitude	The geographic latitude of the location where the animal was released. Units: decimal degrees, WGS84 reference system.
deploy-on-longitude	The geographic longitude of the location where the animal was released. Units: decimal degrees, WGS84 reference system.
deployment-id	A unique identifier for the deployment of a tag on animal.
duty-cycle	Remarks associated with the duty cycle of a tag during the deployment, describing the times it is on/off and the frequency at which it transmits or records data.
manipulation-type	The way in which the animal was manipulated during the deployment. None = The animal received no treatment other than tag attachment and related measurements and sampling.
outlier-comments	A description or reference for methods used to define outliers in 'algorithm marked outlier'.
tag-manufacturer-name	The company or person that produced the tag.
tag-model	The model of the tag.
tag-readout-method	The way the data are received from the tag. satellite = Data are transferred via satellite.

## Figures and Tables

**Table 1.** Satellite tag-derived movements of breeding stock E1 humpback whales. Additional deployment information can be found in Data Resources. Argos PTT = the unique tag identification number; Tracking duration = duration of tag deployment from tag deployment date to last location date; Deploy location = broad geographic location where satellite tag was deployed; Stage of annual cycle upon deployment = migration direction or feeding grounds; Initial activity = whale behaviour at tagging; Retained for SSM = whether the state-space model was applied to the Argos locations generated to account for Argos location error; SSM-derived track distance esimate = the length of the satellite track from the state-space model location estimates in kilometres; Movement captured = the types of movement and behaviour detailed in each satellite track.

**Argos PTT**	**Tracking duration (days)**	**Deploy location**	**Stage of annual cycle upon deployment**	**Initial Activity**	**Retained for SSM**	**SSM derived track distance estimate (km)**	**Movement captured**
96404	1	Antarctica	On feeding grounds	Slow travelling	Yes	68	Antarctic feeding grounds
88752	1	Evans Head, Australia	Migrating north	Travelling	No	NA	NA
53359	1	Sunshine Coast, Australia	Migrating south	Surface active	No	NA	NA
53376	2	Antarctica	On feeding grounds	Feeding	Yes	222	Antarctic feeding grounds
88744	3	Eden, Australia	Migrating south	Feeding	Yes	110	Southern migration along the Australian east coast
53383	3	Antarctica	On feeding grounds	Logging	Yes	244	Antarctic feeding grounds
96401	4	Antarctica	On feeding grounds	Surface active	Yes	68	Antarctic feeding grounds
96395	5	Antarctica	On feeding grounds	Feeding	Yes	219	Antarctic feeding grounds
88743	13	Eden, Australia	Migrating south	Feeding	Yes	535	Southern migration along the Australian east coast
96385	13	Antarctica	On feeding grounds	Fast travelling	Yes	383	Antarctic feeding grounds
88747	13	Evans Head, Australia	Migrating north	Travelling	Yes	901	Northern migration to breeding grounds
64238	14	Sunshine Coast, Australia	Migrating south	Milling	Yes	790	Southern migration along the Australian east coast
96412	15	Antarctica	On feeding grounds	Logging	Yes	663	Antarctic feeding grounds
88736	15	Evans Head, Australia	Migrating north	Travelling	Yes	1016	Northern migration to breeding grounds
88745	18	Eden, Australia	Migrating south	Feeding	Yes	1306	Southern migration towards New Zealand
88746	20	Eden, Australia	Migrating south	Feeding	Yes	1662	Southern migration towards New Zealand
88742	20	Evans Head, Australia	Migrating north	Milling	Yes	1004	Northern migration to breeding grounds
96390	21	Antarctica	On feeding grounds	Surface active	Yes	695	Antarctic feeding grounds
88737	21	Evans Head, Australia	Migrating north	Travelling	Yes	1414	Northern migration to breeding grounds
96403	21	Antarctica	On feeding grounds	Slow travelling	Yes	1680	Antarctic feeding grounds
88751	21	Evans Head, Australia	Migrating north	Travelling	Yes	1334	Northern migration to breeding grounds
88734	26	Evans Head, Australia	Migrating north	Milling	Yes	1376	Northern migration to breeding grounds
88756	26	Evans Head, Australia	Migrating north	Travelling	Yes	1317	Northern migration to breeding grounds then partial southern migration
88750	26	Evans Head, Australia	Migrating north	Milling	Yes	1245	Northern migration to breeding grounds
88753	27	Evans Head, Australia	Migrating north	Travelling	Yes	1064	Northern migration to breeding grounds
88717	29	Eden, Australia	Migrating south	Milling	Yes	1679	Southern migration towards New Zealand
53348	31	Antarctica	On feeding grounds	Feeding	Yes	1107	Antarctic feeding grounds
88732	34	Eden, Australia	Migrating south	Feeding	Yes	2275	Southern migration towards New Zealand
88748	34	Evans Head, Australia	Migrating north	Travelling	Yes	2212	Northern migration to breeding grounds then partial southern migration
88735	38	Eden, Australia	Migrating south	Feeding	Yes	1010	Southern migration along the Australian east coast
88755	39	Evans Head, Australia	Migrating north	Travelling	Yes	1669	Northern migration to breeding grounds then partial southern migration
98138	40	Antarctica	On feeding grounds	Feeding	Yes	1367	Antarctic feeding grounds
98139	40	Sunshine Coast, Australia	Migrating south	Travelling	Yes	2709	Southern migration along the Australian east coast
88733	41	Eden, Australia	Migrating south	Feeding	Yes	3883	Southern migration to Antarctic feeding grounds
88730	44	Evans Head, Australia	Migrating north	Travelling	Yes	2313	Northern migration to breeding grounds
96398	46	Antarctica	On feeding grounds	Logging	Yes	1816	Antarctic feeding grounds
64235	46	Sunshine Coast, Australia	Migrating south	Surface active, moving slowly	Yes	4449	Southern migration to Antarctic feeding grounds
98114	56	Sunshine Coast, Australia	Migrating south	Travelling	Yes	4600	Southern migration to Antarctic feeding grounds
88738	57	Eden, Australia	Migrating south	Travelling	Yes	4099	Southern migration to Antarctic feeding grounds
88754	58	Evans Head, Australia	Migrating north	Travelling	Yes	3117	Northern migration to breeding grounds then partial southern migration
98109	65	Antarctica	On feeding grounds	Slow travelling	Yes	442	Antarctic feeding grounds
88725	80	Eden, Australia	Migrating south	Unknown	Yes	4303	Southern migration to Antarctic feeding grounds
88723	81	Eden, Australia	Migrating south	Feeding	Yes	5321	Southern migration to Antarctic feeding grounds
88718	91	Eden, Australia	Migrating south	Feeding	Yes	5050	Southern migration to Antarctic feeding grounds
96386	92	Antarctica	On feeding grounds	Slow travelling	Yes	3805	Antarctic feeding grounds
88728	92	Eden, Australia	Migrating south	Unknown	Yes	5540	Southern migration to Antarctic feeding grounds
88729	98	Eden, Australia	Migrating south	Feeding	Yes	6352	Southern migration to Antarctic feeding grounds
98129	104	Sunshine Coast, Australia	Migrating south	Unknown	Yes	6636	Southern migration to Antarctic feeding grounds
88741	154	Eden, Australia	Migrating south	Travelling	Yes	8580	Southern migration towards New Zealand and then onto Antarctic feeding grounds
98100	286	Sunshine Coast, Australia	Migrating south	Travelling	Yes	7046	Partial southern migration then northern migration to breeding grounds following an 8 month gap in data transmission

**Table 2.** Monthly track summary detailing the number of tracks occurring in that month, the number of state space modelled location estimates generated by those tracks, the mean latitude of the location estimates and the dominant direction of travel.

**Month**	**Number of locations**	**Individual tracks**	**Mean latitude**	**Dominant direction of travel**
**Jan**	1873	7	63.9°S	6 x south, 1 x north
**Feb**	739	9	65.7°S	1 x south, 5 x resident, 3 x north
**Mar**	2476	12	66.2°S	1 x south, 1 x north, 10 x resident
**Apr**	737	5	64.4°S	3 x north, 2 x resident
**May**	356	1	64.0°S	1 x resident
**Jun**	265	13	27.1°S	13 x north
**Jul**	742	14	22.6°S	10 x north, 3 x north then south
**Aug**	119	3	27.6°S	1 x north then south, 2 x south
**Sept**	0	0	NA	NA
**Oct**	910	18	34.7°S	4 x resident, 12 x south
**Nov**	2881	19	44.6°S	3 x resident, 16 x south
**Dec**	2213	12	58.7°S	10 x south, 2 x data limited
